# Internet Gaming Disorder and Internet Addiction: Comparing Italian and Migrant Children and Adolescents

**DOI:** 10.3390/pediatric18020053

**Published:** 2026-04-07

**Authors:** Giovanni Giulio Valtolina, Diego Boerchi, Luca Milani

**Affiliations:** 1 Department of Psychology, Catholic University of the Sacred Heart, 20121 Milan, Italy; diego.boerchi@unicatt.it (D.B.); luca.milani@unicatt.it (L.M.); 2 WWELL Research Center, Catholic University of the Sacred Heart, 20121 Milan, Italy; 3 Iniziative e Studi sulla Multietnicità (ISMU) Foundation, 20125 Milan, Italy; 4 Centro di Ricerca sulle Dinamiche Evolutive ed Educative (C.Ri.d.e.e.), Catholic University of the Sacred Heart, 20121 Milan, Italy

**Keywords:** internet addiction, internet gaming disorder, migration, developmental risk, coping strategies, interpersonal relationships

## Abstract

Background: research suggests that adolescents with a migrant background may be particularly vulnerable to behavioral addictions, including problematic gaming and Internet use. Methods: we compared Italian (ITA) and non-Italian (WIC) students on Internet Gaming Disorder (IGD) and Internet Addiction (IA) and examined whether coping strategies and interpersonal-relationship quality were associated with these outcomes, using robust linear models estimated with the GENLIN procedure in IBM SPSS Statistics 31 and regression-based models on observed variables. A total of 535 students (64.5% female; aged 9–18) completed the Video Games Addiction Questionnaire (VGA), the Internet Addiction Test (IAT), the Children’s Coping Strategies Checklist–Revised (CCSC), and the Assessment of Interpersonal Relations (AIR). Results: robust generalized linear models showed that WIC adolescents reported significantly higher IGD levels than their Italian peers, while no differences emerged for IA. Gender differences were evident only in unadjusted models, with males reporting higher IGD and females higher IA; however, these effects were not significant once age and nationality were considered simultaneously. Age was positively associated with IA but not with IGD. Avoidance coping was associated with higher levels of both IGD and IA, whereas active coping was negatively associated with IGD. Relationship quality was not associated with IGD but showed protective effects for IA: better relationships with mothers and with both male and female peers were associated with lower IA scores. Overall, the findings highlight that IGD and IA follow partially distinct developmental patterns. Migrant background emerged as a specific vulnerability factor for IGD, while IA appears more closely linked to age-related processes, coping styles, and interpersonal-relationship quality. Conclusions: the results call for differentiated prevention and intervention approaches targeting the distinct etiological mechanisms of each problematic behavior, focusing on coping and migration-related stress and belonging for IGD, and on strengthening coping repertoires and relational resources for IA.

## 1. Introduction

In 2023, about 5.3 billion people connected to the Internet, which is equivalent to 66% of the global population. This number is only set to increase as we move forward in 2026. Of this total, 5.04 billion, or 62.3% of the world’s population, were social media users [1]. Over the last 15 years, research on new technology addiction has increased exponentially, but the phenomenon of Internet Addiction (IA) is not yet unequivocally outlined because there is no single, agreed-upon definition or set of diagnostic criteria, and different models exist with varying symptoms and emphases [2]. Currently, IA is not included as an officially recognized disorder in the DSM-5-TR. Instead, the focus is mainly on Internet Gaming Disorder (IGD), a condition related to IA, included in the DSM-5-TR as a “condition for further study” [3].

Due to the widespread use of the Internet, especially among young people, research has focused on adolescents, stressing individual and relational correlates. More specifically, the most at-risk youngsters show low social openness, reduced emotional intelligence and a liking for secluded activities [4,5]. Other correlates are a high level of perceived loneliness [6] and the presence of depressive traits with suicidal ideation [7,8]. In this respect, Caplan [9] introduced the term “Preference for Online Social Interaction” (POSI) to describe adolescents perceiving themselves as safer, more effective, more confident and more comfortable in online interaction than in traditional face-to-face interactions. A more recent study investigated the relationship between social anxiety, motivation, and POSI and suggested that metacognitions may play an important role in the association between social anxiety and Internet Gaming Disorder (IGD), along with POSI [10]. Moreover, permissive parenting style can be included in environmental correlates [11].

In terms of developmental outcomes, the disorder has been associated with the following negative features: identity shortfalls [12], brain structure alterations [13,14], impairments in cognitive functioning [15], risk-seeking behavior [16,17], low quality of interpersonal relationships [18,19], eating disorders [20], internalizing symptoms [21,22] and self-harming behavior [23,24].

Research investigating the prevalence of IGD shows it is higher for males [25] and it is more widespread in Asian countries [26]. The prevalence appears to range between 3.4% and 6.4%, with a high discrepancy between males (8.4–15%) and females (2.9–6.9%) [26].

Although the time spent gaming is a potential correlate for the development of addiction, so far, this is not a diagnostic criterion. There is a consensus that addicted gamers spend more time gaming than the control population [27,28]. However, more than the time itself, a more relevant correlate seems to be the gaming habits, i.e., gaming sessions at unusual times of the day and at night during the working week [29,30].

Regarding consequences of IGD in terms of maladaptive outcomes, the literature shows that youths at risk of gaming addiction tend to prefer mediated and virtual relationships over face-to-face ones [31] and are characterized by behavioral problems [32], ADHD [33,34], low performance at school [35,36], and risk of alcohol and substance abuse [37,38].

The significance of ethnicity in the development of migrant children is substantial [39]. Ethnic belonging is related to all physiological and psychological changes in human development, mainly affecting family and peer relationships [40]. So, although with significant differences depending on several variables (place of birth, country of origin, integration, family aim, etc.), children with a migratory background are thought to be at significant developmental risk, with psychological distress and risk-taking behaviors, such as smoking, drinking, unsafe sex, drug addiction [41,42], and IGD [43,44]. Although nosological classification of this phenomenon is still a matter of debate, it is argued that IGD might be described as a non-substance-related addiction.

The purpose of this work is to collect preliminary data on the prevalence of technological addictions in a population of students with a migratory background compared to Italian ones and to identify possible maladaptive correlates of the disorders. For the research, we decided to verify both the prevalence and characteristics of IGD—a diagnosis consolidated in the literature and included in the DSM-5-TR in the section recommending conditions for further research—and the Internet Addiction construct, as it is still widely considered even in the academic field.

The study’s aims can be summarized as follows: testing the differences between Italian and migrant students in terms of problematic use of video games and the Internet; identifying any correlates associated with the emergence of problematic involvement in video game activities and Internet use. To investigate both the differences in the distribution of addictions between students with Italian citizenship (ITA) and those without Italian citizenship (WIC), as well as between males and females, and the role coping strategies and interpersonal relationships can play in increasing addiction to video games and the Internet, we tested the following hypotheses:

(1) WIC students’ scores in terms of IGD and Internet Addiction are higher than ITA students’ scores;
(2) Mmale students’ scores for IGD are higher than female students’ scores, while female students’ scores for Internet Addiction will be higher than male students’ scores;
(3) Male and female students’ scores for IGD and Internet Addiction are not different regarding their citizenship;
(4) Both IGD and Internet Addiction are positively associated with age;
(5) Maladaptive coping strategies are positively associated with both IGD and Internet Addiction;
(6) The quality of interpersonal relationships is negatively associated with IGD and Internet Addictions.

Despite growing research on adolescent digital risks, migrant-origin youth remain underrepresented, and evidence on differential mechanisms for IGD vs. IA is mixed. Focusing on Italian (ITA) and non-Italian (WIC) students addresses this gap and offers preliminary, context-sensitive evidence to inform larger studies.

## 2. Materials and Methods

### 2.1. Participants

Given the small WIC subgroup and the within-school recruitment, all between-group contrasts should be considered preliminary.

A total of 552 students participated in the study. After screening multivariate outliers using the Mahalanobis distance, 17 students (3.1%) were excluded, resulting in a final analytic sample of 535 participants. Age ranged from 9 to 18 years old (M = 15.80; SD = 2.161) attending different types and orders of schools in four big Italian cities (Brescia, Milano, Roma and Verona). Gender was not equally distributed, with females more represented than males (F = 64.5%; M = 35.5%). Fifty-three students (9.9% of the analytic sample) had no Italian citizenship, with 18 originating in European and 35 in non-European countries. We preferred a smaller WIC group recruited within the same school context as the ITA group, rather than enlarging the WIC group by adding students from different schools, which would have reduced contextual comparability and introduced potential confounding due to school- and area-level differences. Consistent with this design choice, ITA and WIC did not differ in age (t(533) = −1.383, *p* = 0.167, Hedges’ g = −0.20, 95% CI [−0.48, 0.08]) or gender (χ^2^(1) = 0.042, *p* = 0.881, φ = 0.009), supporting basic baseline comparability between groups.

### 2.2. Measures

Justification for the IAT: We used the Internet Addiction Test (IAT) because it remains widely adopted and psychometrically studied in adolescent research, enabling comparability with prior evidence. Nevertheless, the IAT captures broad Internet use and may overlap with social media-oriented behaviors; we acknowledge these limitations.

Why the GPIUS was not included: To minimize respondent burden in classroom settings and to prioritize coping and interpersonal measures aligned with our aims, we did not administer Caplan’s GPIUS. We note this as a limitation and a priority for future research.

Terminology note: We consistently use ‘Internet Gaming Disorder (IGD)’ for the construct and retain ‘Video Games Addiction’ only when referring to the instrument name (VGA).

Participants completed the following questionnaires:

- *Revised Video Games Addiction Questionnaire* (VGA) [45], composed of 16 items on a 3-point Likert scale, aimed at identifying the possible presence of problematic and uncontrolled use of video games (both online and offline).
- *Internet Addiction Test* (IAT) [46], composed of 20 items on a 5-point Likert scale, aimed at investigating how the use of the Internet can affect social life, academic and job career quality, and time control.
- *Children’s Coping Strategies Checklist* (CCSC) [47,48], composed of 54 items on a 4-point Likert scale, aimed at testing children and adolescents’ ability to cope with stress by means of the use of cognitive coping strategies (active coping, distraction strategies, avoidance strategies, support-seeking strategies).
- *Assessment of Interpersonal Relations* (AIR) [49], composed of five subscales (relationship with the mother, with the father, with the female peers, with the male peers and with the teachers) with the same 35 items on a 4-point Likert scale; it measures the interpersonal competence of adolescents in relationships with peers and with adult figures.

### 2.3. Procedure

Parental consent and student assent. Consent was obtained from both parents or from the legal guardian in accordance with school policies and Ethics Committee guidance; when only one parent held legal custody, that signature sufficed. All students provided active assent at administration and could decline or discontinue without penalty.

The study was presented to the students indicating that its goal was to investigate their behaviors related to video games and the use of the Internet, and how they deal with life events and people closest to them. Subsequently, their parents were given a letter of presentation of the study and a form to collect their informed consent signed by both parents. The questionnaire’s administration to the students took place in group mode in the classrooms and during school hours. Participation was voluntary, and participants were informed of the possibility of withdrawing from the collaboration at any time. Data were collected prior to the COVID-19 pandemic. Although data were collected pre-pandemic, the phenomenon has only increased in salience; we therefore treat the context as conservative.

### 2.4. Data Analysis

Given the imbalanced subgroup sizes, the mediation analyses were exploratory and based on observed variables with bootstrap CIs; no latent multigroup models were estimated.

To test Hypotheses 1–4, the associations with IGD and IA were examined separately using robust linear models estimated with the GENLIN procedure in IBM SPSS Statistics 31 (normal distribution, identity link). Nationality, gender, and their interaction were included to assess whether the association with the outcomes differed across nationality and gender groups. Age was included as a covariate, and its main effect was used to evaluate Hypothesis 4. Nationality was coded 0 = ITA and 1 = WIC; gender was coded 0 = male and 1 = female. Effects are reported as unstandardized coefficients (B) with 95% confidence intervals; standard errors were estimated using heteroskedasticity-consistent (robust) procedures. Robust methods mitigate departures from normality, and we aimed to prioritize the interpretability of unstandardized effects.

To verify whether gender exhibited raw associations with IGD and IA prior to statistical adjustment, an aspect relevant to Hypothesis 2, we additionally estimated unadjusted robust GLMs including gender as the only covariate. The purpose was to determine whether any gender differences observed at the bivariate level remained when age, nationality, and coping strategies were subsequently included in the adjusted analyses.

Hypotheses 5–6 were examined using regression-based models on observed variables. For Hypothesis 5, coping strategies were entered simultaneously in models, showing association with IGD/IA while controlling for age, gender, and nationality group, to estimate the unique contribution of each coping strategy net of the others. In addition, an exploratory parallel multiple-mediation model (PROCESS v5, model 4) was estimated to examine whether nationality differences in IGD/IA were indirectly associated with coping strategies, using nonparametric bootstrap confidence intervals for indirect effects. For Hypothesis 6, interpersonal-relationship quality was primarily conceptualized as a global construct; therefore, it was tested using a composite index. To facilitate interpretation of domain-specific associations and to minimize collinearity-driven suppression among correlated relationship indicators, each relationship quality indicator was also examined in one-at-a-time models showing associations with IGD/IA while controlling for the same covariates, with heteroskedasticity-consistent (robust) standard errors. Given the cross-sectional design, all findings were interpreted as associational rather than causal.

## 3. Results

Robust GLMs were estimated separately for IGD and IA, including nationality, gender, their interaction, and age as covariates. For IGD, the main effect of nationality was significant, Wald χ^2^(1) = 7.25, *p* = 0.007, indicating higher IGD scores in the WIC group than in the ITA group; gender, the nationality × gender interaction, and age were not significant (Table 1). Consistent with this effect, the adjusted WIC–ITA difference among females was 0.80 points (95% CI [0.11, 1.48]).

For IA, age was positively associated with IA scores, Wald χ^2^(1) = 19.71, *p* < 0.001 (Table 1). Nationality, gender, and their interaction were not significant. Adjusted marginal means by nationality and gender are reported in Table 2.

Parallel multiple-mediation models (PROCESS v5, model 4) were estimated on observed variables to explore whether coping strategies were indirectly associated with the relationship between nationality group and (a) IGD and (b) IA. Coping strategies were entered simultaneously as mediators. Age and gender were included as covariates. Indirect effects were evaluated using 5000 bias-corrected bootstrap samples and 95% confidence intervals.

For IGD, nationality group showed a significant total effect, B = 0.729, SE = 0.202, t = 3.61, *p* < 0.001, 95% CI [0.332, 1.126], indicating higher IGD scores in the WIC group. The direct effect remained significant after accounting for coping strategies, B = 0.673, SE = 0.199, t = 3.38, *p* < 0.001, 95% CI [0.282, 1.063] (Table 3). The total indirect effect through coping was not significant (B = 0.057, boot 95% CI [−0.062, 0.185]), and none of the specific indirect effects reached significance (all boot 95% CIs included zero; Table 4). In the outcome equation, active coping was negatively associated with IGD and avoidance coping was positively associated with IGD; moreover, after accounting for these coping dimensions, nationality remained significantly associated with IGD (Table 3), consistent with H5.

For IA, nationality group showed neither a significant total effect, B = 0.217, SE = 2.119, t = 0.10, *p* = 0.918, 95% CI [−3.946, 4.380], nor a significant direct effect after accounting for coping strategies, B = −0.544, SE = 2.088, t = −0.26, *p* = 0.794, 95% CI [−4.646, 3.557] (Table 3). The total indirect effect through coping was not significant (B = 0.762, boot 95% CI [−0.430, 2.212]), and none of the specific indirect effects were significant (Table 4). In the IA outcome model, age and gender were significant covariates, and avoidance coping was positively associated with IA (Table 3), consistent with H5.

Although the adjusted GLMs used to evaluate the main hypotheses did not identify gender as associated with IGD or IA (Table 1), gender differences clearly emerged under certain model specifications (Table 3). Then, we decided to examine gender in isolation, and males reported significantly higher IGD scores than females (Wald χ^2^(1) = 13.35, *p* < 0.001; estimated means = 1.64 vs. 1.16), whereas females reported significantly higher IA scores than males (Wald χ^2^(1) = 10.62, *p* = 0.001; estimated means = 33.72 vs. 29.35). In summary, gender effects appeared when tested in isolation, disappeared once age and nationality were added to the model, and re-emerged when coping strategies were included as additional covariates.

In robust GLMs testing H6, relationship quality was not associated with IGD. The composite relationship quality index showed no association with IGD (B = 0.000, 95% CI [−0.007, 0.008], *p* = 0.959), and none of the relationship domains were significant when entered separately (Table 5).

For IA, the composite index showed a marginal negative association (B = −0.092, 95% CI [−0.186, 0.001], *p* = 0.053). In one-variable-at-a-time models controlling for age, gender, and nationality group, higher relationship quality with the mother (B = −0.072, 95% CI [−0.130, −0.013], *p* = 0.017), with male peers (B = −0.081, 95% CI [−0.147, −0.015], *p* = 0.016), and with female peers (B = −0.110, 95% CI [−0.172, −0.049], *p* < 0.001) was associated with lower IA. Relationship quality with the father (B = −0.047, 95% CI [−0.105, 0.010], *p* = 0.108), and the teachers (B = 0.061, 95% CI [−0.004, 0.126], *p* = 0.064) was not significant (Table 5). Overall, H6 was not supported for IGD and was partially supported for IA.

## 4. Discussion

Measurement notes on gender differences: Several IAT items emphasize time management and interpersonal interference, which may map more closely onto communication- and social media-oriented use. This content coverage could partly contribute to observed gender patterns. Future studies should include domain-specific measures to improve precision.

WIC estimates should be interpreted cautiously due to limited power and wide confidence intervals.

The present study aimed to deepen the understanding of Internet Gaming Disorder (IGD) and Internet Addiction (IA) among Italian adolescents and peers with a migrant background, while also examining the role of coping strategies and interpersonal-relationship quality. In doing so, it speaks to current developmental models that view problematic digital engagement as the outcome of an interaction between individual vulnerabilities, affective–cognitive processes, and environmental context (e.g., stress exposure and available offline resources). The findings provide a nuanced picture, highlighting both shared and divergent developmental pathways for IGD and IA, and confirming only some of the theoretical expectations derived from the previous literature.

Consistent with Hypothesis 1, adolescents without Italian citizenship reported significantly higher levels of IGD compared to their Italian peers. This result aligns with previous evidence [50,51] suggesting that migrant-origin adolescents may face heightened developmental risks due to stressors linked to acculturation, identity negotiation, and social integration challenges, which may amplify vulnerability toward behavioral addictions, including gaming disorders. Research has emphasized that ethnicity and migratory experiences can shape both psychological distress and risk-taking behaviors across adolescence, including substance use, gambling, and problematic digital engagement. The fact that these differences emerged for IGD but not for IA is particularly noteworthy. IA did not differ between the two groups, suggesting that certain digital behaviors—such as problematic Internet use more generally—might reflect widespread adolescent patterns that transcend cultural and migratory divides, whereas gaming behaviors could be more sensitive to specific stressors related to cultural transition or social belonging.

One plausible interpretation is that gaming may function as a low-barrier space for emotion regulation and social compensation when offline opportunities are constrained by language barriers, discrimination experiences, reduced extracurricular access, or fragmented peer networks. At the same time, “without Italian citizenship” is an administrative proxy that does not capture key migration-related dimensions (e.g., generation status, length of residence, language proficiency, perceived discrimination, socioeconomic hardship), which should be measured directly in future work to clarify the mechanisms behind the observed IGD difference.

Hypothesis 2 received partial support. As widely documented in the prior literature [52,53], males tend to report higher gaming involvement and are generally more susceptible to IGD, while females typically report higher levels of IA—often associated with social or emotional regulation motives. These patterns emerged clearly when gender was examined in isolation: males scored higher on IGD, whereas females scored higher on IA. Yet these effects vanished in the primary adjusted models, suggesting that gender differences interact with age, citizenship, and coping strategies in complex ways. One statistical possibility is suppression: if age, nationality composition, and coping covary with gender in the sample, the “pure” gender coefficient can shrink or change sign when covariates are introduced. Substantively, the finding is also compatible with a pathway view in which gender differences reflect partly different patterns of digital activities (e.g., game genres versus social or messaging uses) and motives (achievement/competition versus social connection or affect regulation), rather than a simple male–female gap in “addiction proneness”. This instability echoes prior findings showing that gender effects in digital addictions can shift depending on contextual and psychological covariates, reflecting the heterogeneity of pathways leading to problematic technology use. The re-emergence of gender differences once coping strategies were included indicates that part of the gender effect may be embedded in gender-differentiated coping styles, a point discussed further below.

Hypothesis 3, forecasting no interaction between gender and citizenship, was fully supported. The absence of a significant Nationality × Gender interaction confirms that the differences observed between males and females do not depend on whether the adolescent has a migrant background. This finding reinforces the notion that the two variables represent independent dimensions of developmental risk or protection. This is an important null finding because it suggests that prevention messages need not assume qualitatively different gender-by-migration risk profiles, at least when migration is operationalized via citizenship and within the sampled school contexts. However, this null interaction should be interpreted cautiously, as interaction tests are typically underpowered in small subgroups. Given the limited size of the WIC group (*n* = 53), modest moderation effects cannot be ruled out.

Turning to Hypothesis 4, the results reveal a clear divergence between IGD and IA. Age was not significantly associated with IGD, suggesting that gaming involvement—and even problematic gaming—may be less developmentally linear than expected. In contrast, age was significantly and positively associated with IA, confirming that older adolescents are more prone to problematic Internet use. This trend is consistent with prior studies [54,55], indicating that as adolescents grow older, digital engagements increasingly become intertwined with identity processes, autonomy seeking, and emotional coping, which may heighten vulnerability to maladaptive Internet use but not necessarily to gaming-related problems. Given that data were collected prior to the COVID-19 pandemic, it will be important to test whether the age gradient for IA has strengthened (or shifted earlier) in more recent cohorts exposed to sustained remote schooling and higher baseline screen time. Overall, IGD may therefore be influenced by age-neutral factors such as gaming culture, peer dynamics, or personality traits, whereas IA may reflect developmental trajectories more directly linked to psychosocial maturation.

Hypothesis 5 received strong empirical support and represents one of the most robust findings of the study. Coping strategies, particularly maladaptive ones, were consistently associated with both IGD and IA [56], as the literature underlines. Avoidant coping emerged as positively associated with both outcomes, highlighting its role as a correlate. Conversely, active coping was associated with lower IGD, suggesting a protective effect. These findings are—as stated earlier—highly consistent with a large body of the literature [57,58] showing that adolescents who rely on avoidance strategies to manage stress are more likely to develop problematic digital behaviors, often using online environments as implicit emotion regulation tools. From an applied standpoint, this pattern points to prevention targets that are more realistic than “reduce screen time” slogans: strengthening coping repertoires (problem-solving, emotion labeling, help-seeking) and reducing avoidant cycles may directly lower risk for both outcomes, even when structural stressors remain. The absence of indirect effects in the mediation models indicates that coping strategies do not explain the higher levels of IGD found among WIC students. This suggests that other mechanisms—possibly related to belonging, discrimination, family migration stress, or differential opportunities for offline leisure—might contribute to the observed differences.

Finally, Hypothesis 6 was only partially supported. Relationship quality showed no significant association with IGD, contradicting the expectation based on previous research suggesting that low interpersonal-relationship quality is often linked with heightened gaming involvement [59]. One interpretation is that contemporary gaming can also be socially embedded (cooperative play, guilds, voice chat), so high engagement is not necessarily a marker of interpersonal withdrawal and may even coexist with satisfactory offline relationships. Another possibility is measurement specificity: the AIR focuses on offline relationships and may not capture online peer bonding, parental digital mediation, or family conflict around gaming, which could be more proximal to IGD. The absence of such effects may reflect the more structured or goal-oriented nature of gaming activities compared to general Internet use. In contrast, IA was significantly associated with relationship quality in several relational domains. More positive relationships with mothers and both male and female peers were associated with lower IA levels. This pattern supports longstanding evidence that strong interpersonal bonds and secure relational environments function as protective buffers against maladaptive digital behaviors [60,61]. Interestingly, relationships with fathers and teachers did not show significant associations, perhaps pointing to the particular importance of maternal and peer relationships in the everyday socio-emotional regulation processes of adolescents. The marginal significance of the composite relationship index further supports the idea that IA—more than IGD—is intertwined with the quality of adolescents’ relational ecosystem [62].

Taken together, these findings underline the importance of distinguishing between IGD and IA as partially separate phenomena. Although they share common correlates such as avoidant coping, they also display distinct patterns: IGD is more sensitive to citizenship background and less tied to relational or developmental trajectories, whereas IA appears more deeply embedded in age-related processes and interpersonal dynamics. This conceptual separation aligns with contemporary debates in the literature questioning the overlap between different forms of technological addictions and advocating for more nuanced, domain-specific models. For practice, the results suggest differentiated prevention: for IA, relational strengthening and peer connection (especially with mothers and close peers) may be central; for IGD, interventions may need to focus more on coping and on migration-related stress and belonging, rather than assuming poor relationship quality as the primary driver.

## 5. Limitations and Future Directions

Several limitations should be considered. First, the design is cross-sectional, so the observed associations cannot be interpreted causally, and bidirectional processes are plausible (e.g., avoidant coping may increase problematic use, but problematic use may also erode coping resources).

Second, the WIC group is relatively small and heterogeneous (European vs. non-European origins) and citizenship is an imperfect proxy for migratory background; future work should include larger samples and directly assess generation status, length of residence, language proficiency, socioeconomic position, and perceived discrimination.

Third, all measures are self-reported and do not include objective indicators (time online, game genre, night-time use) or multi-informant perspectives (parents/teachers), which could reduce shared-method variance and clarify context.

Fourth, data were collected prior to the COVID-19 pandemic; cohort effects in digital habits and normative exposure may limit generalizability to current adolescents.

Despite these limitations, the study contributes preliminary evidence that a migrant background may be a specific vulnerability factor for IGD, whereas IA appears more tightly linked to developmental age processes, avoidant coping, and relational quality. These distinctions support the need for targeted, mechanism-informed prevention approaches.

Future studies should (a) directly measure migration-related experiences beyond citizenship (acculturative stress, discrimination, language competence), (b) test measurement invariance of the instruments across groups, and (c) use longitudinal designs to disentangle selection versus socialization processes and clarify temporal ordering among coping, relationships, and problematic use.

For causal inference, future work should (a) use cross-lagged panel models to test temporal precedence among coping, relationships, and IGD/IA, and (b) implement randomized, school-based coping-skills interventions with pre/post and follow-up assessments of IGD/IA.

## 6. Conclusions

IGD and IA showed partly distinct patterns: migrant background was specifically associated with higher IGD, whereas IA related more strongly to age, avoidant coping, and interpersonal-relationship quality. Prevention should therefore be differentiated: for IGD, address coping and migration-related stress and belonging; for IA, strengthen coping repertoires and relational resources. Findings for the WIC subgroup are preliminary and call for larger, multi-informant, and longitudinal studies.

## Figures and Tables

**Table 1:** Robust GLM (Type III Wald tests) for IGD and IA.

Effect	IGD Wald χ^2^(1)	IGD *p*	IA Wald χ^2^(1)	IA *p*
Nationality	7.25	0.007	0.06	0.805
Gender	2.15	0.143	1.14	0.285
Nationality × Gender	0.11	0.735	0.40	0.529
Age	1.80	0.180	19.71	<0.001

**Table 2:** Adjusted marginal means (95% CI) by nationality and gender (age held constant at the model value).

Group	Gender	IGD (Adjusted Mean, 95% CI)	IA (Adjusted Mean, 95% CI)
ITA	Male	1.57 [1.34, 1.80]	29.54 [27.40, 31.68]
ITA	Female	1.09 [0.95, 1.22]	33.58 [31.96, 35.20]
WIC	Male	2.18 [1.44, 2.93]	31.61 [24.62, 38.60]
WIC	Female	1.88 [1.21, 2.55]	32.67 [27.18, 38.16]

**Table 3:** Regression-based mediation models (outcome equations). Unstandardized coefficients (B) with 95% CI.

	B	SE	t	*p*	95% CI
IGD					
Nationality Group (direct; c′)	0.673	0.199	3.38	<0.001	[0.282, 1.063]
Active Coping	−0.373	0.159	−2.34	0.020	[−0.686, −0.060]
Distraction Coping	0.123	0.119	1.04	0.300	[−0.110, 0.356]
Avoidance Coping	0.730	0.151	4.83	<0.001	[0.433, 1.026]
Support Seeking Coping	0.119	0.104	1.15	0.250	[−0.084, 0.322]
Age	−0.032	0.028	−1.15	0.251	[−0.086, 0.022]
Gender	−0.531	0.125	−4.26	<0.001	[−0.776, −0.286]
IA					
Nationality Group (direct; c′)	−0.544	2.088	−0.26	0.794	[−4.646, 3.557]
Active Coping	−2.913	1.673	−1.74	0.082	[−6.200, 0.374]
Distraction Coping	0.743	1.244	0.60	0.551	[−1.702, 3.188]
Avoidance Coping	7.992	1.585	5.04	<0.001	[4.878, 11.106]
Support Seeking Coping	0.403	1.087	0.37	0.711	[−1.731, 2.537]
Age	1.337	0.289	4.63	<0.001	[0.769, 1.904]
Gender	3.077	1.309	2.35	0.019	[0.505, 5.649]

**Table 4:** Indirect effects of nationality group on outcomes through coping strategies.

	Effect	BootSE	Boot 95% CI
IGD			
Total Indirect	0.057	0.062	[−0.062, 0.185]
Via Active Coping	−0.020	0.029	[−0.104, 0.021]
Via Distraction Coping	0.015	0.021	[−0.008, 0.094]
Via Avoidance Coping	0.082	0.055	[−0.012, 0.211]
Via Support Seeking Coping	−0.020	0.021	[−0.084, 0.008]
IA			
Total Indirect	0.762	0.665	[−0.430, 2.212]
Via Active Coping	−0.154	0.253	[−0.951, 0.156]
Via Distraction Coping	0.089	0.193	[−0.147, 0.697]
Via Avoidance Coping	0.894	0.626	[−0.126, 2.382]
Via Support Seeking Coping	−0.068	0.210	[−0.621, 0.277]

**Table 5:** Relationship quality associations with IA and IGD (separate one-variable-at-a-time models controlling for age, gender, and nationality group).

	IA		IGD	
	B [95% CI]	*p*	B [95% CI]	*p*
Relationship quality index	−0.092 [−0.186, 0.001]	0.053	0.000 [−0.007, 0.008]	0.959
Relationship with the mother	−0.072 [−0.130, −0.013]	0.017	0.002 [−0.003, 0.007]	0.351
Relationship with the father	−0.047 [−0.105, 0.010]	0.108	0.003 [−0.003, 0.008]	0.335
Relationship with male peers	−0.081 [−0.147, −0.015]	0.016	−0.003 [−0.009, 0.003]	0.345
Relationship with female peers	−0.110 [−0.172, −0.049]	0.000	−0.001 [−0.006, 0.005]	0.811
Relationship with teachers	0.061 [−0.004, 0.126]	0.064	−0.002 [−0.008, 0.004]	0.566

## Data Availability

The raw data supporting the conclusions of this article will be made available by the authors on request. The data are not publicly available due to privacy restrictions.
